# Supplementary material for: HIVprotI: an integrated web based platform for prediction and design of HIV proteins inhibitors
Source: J Cheminform. 2018 Mar 9;10:12. doi: 10.1186/s13321-018-0266-y (PMC5845081; doi:10.1186/s13321-018-0266-y)
Supplement: Supplementary file 2 — Additional file 2. Source code of HIVProtI web server. [file 13321_2018_266_MOESM2_ESM.zip › HIVprotI_Source-code/hivprotI/weka-3-6-6/doc/weka/associations/package-summary.html]

weka.associations


---


|  |  |  |
| --- | --- | --- |
| **Overview** \| **Package** \| Class \| **Tree** \| **Deprecated** \| **Index** \| **Help** \| **Weka's home** | |  |
| PREV PACKAGE   **NEXT PACKAGE** | **FRAMES**    **NO FRAMES**     **All Classes** |  |


---

## Package weka.associations

| **Interface Summary** | |
| --- | --- |
| **Associator** |  |
| **CARuleMiner** | Interface for learning class association rules. |

| **Class Summary** | |
| --- | --- |
| **AbstractAssociator** | Abstract scheme for learning associations. |
| **Apriori** | Class implementing an Apriori-type algorithm. |
| **AprioriItemSet** | Class for storing a set of items. |
| **AssociatorEvaluation** | Class for evaluating Associaters. |
| **CaRuleGeneration** | Class implementing the rule generation procedure of the predictive apriori algorithm for class association rules. |
| **CheckAssociator** | Class for examining the capabilities and finding problems with associators. |
| **FilteredAssociator** | Class for running an arbitrary associator on data that has been passed through an arbitrary filter. |
| **FPGrowth** | Class implementing the FP-growth algorithm for finding large item sets without candidate generation. |
| **FPGrowth.AssociationRule** |  |
| **FPGrowth.BinaryItem** | Inner class that handles a single binary item |
| **GeneralizedSequentialPatterns** | Class implementing a GSP algorithm for discovering sequential patterns in a sequential data set.  The attribute identifying the distinct data sequences contained in the set can be determined by the respective option. |
| **ItemSet** | Class for storing a set of items. |
| **LabeledItemSet** | Class for storing a set of items together with a class label. |
| **Messages** | Messages. |
| **PredictiveApriori** | Class implementing the predictive apriori algorithm to mine association rules.  It searches with an increasing support threshold for the best 'n' rules concerning a support-based corrected confidence value.    For more information see:    Tobias Scheffer: Finding Association Rules That Trade Support Optimally against Confidence. |
| **PriorEstimation** | Class implementing the prior estimattion of the predictive apriori algorithm for mining association rules. |
| **RuleGeneration** | Class implementing the rule generation procedure of the predictive apriori algorithm. |
| **RuleItem** | Class for storing an (class) association rule. |
| **SingleAssociatorEnhancer** | Abstract utility class for handling settings common to meta associators that use a single base associator. |
| **Tertius** | Finds rules according to confirmation measure (Tertius-type algorithm).    For more information see:    P. |

| **Enum Summary** | |
| --- | --- |
| **FPGrowth.AssociationRule.METRIC\_TYPE** | Enum for holding different metric types |

---


|  |  |  |
| --- | --- | --- |
| **Overview** \| **Package** \| Class \| **Tree** \| **Deprecated** \| **Index** \| **Help** \| **Weka's home** | |  |
| PREV PACKAGE   **NEXT PACKAGE** | **FRAMES**    **NO FRAMES**     **All Classes** |  |


---
